# Oral Leukoplakia Microbiome Predicts the Degree of Dysplasia and is Shaped by Smoking and Tooth Loss

**DOI:** 10.1111/odi.15272

**Published:** 2025-02-04

**Authors:** Sheila Galvin, Bahman Honari, Sviatlana Anishchuk, Claire M. Healy, Gary P. Moran

**Affiliations:** ^1^ Division of Oral and Maxillofacial Surgery, Oral Medicine and Oral Pathology Dublin Dental University Hospital and School of Dental Science, Trinity College Dublin Dublin Republic of Ireland; ^2^ Division of Oral Biosciences Dublin Dental University Hospital and School of Dental Science, Trinity College Dublin Dublin Republic of Ireland

**Keywords:** dysplasia, microbiome, oral cancer, oral leukoplakia, smoking

## Abstract

**Objective:**

This study aimed to determine if the oral potentially malignant disorder, oral leukoplakia (OLK), exhibited microbiome changes that predict the degree of dysplasia and the risk of malignant progression.

**Results:**

We examined the microbiome in 216 swabs of OLK from 177 patients. Compared to healthy controls (*n* = 120 swabs from 61 patients), who were less likely to smoke and had better oral health, OLK patients exhibited an increased abundance of *Rothia mucilaginosa* , *Streptococcus parasanguinis* and *S. salivarius,* resembling acetaldehyde generating communities described previously. Compared to the patients' healthy contralateral normal (CLN) mucosa (*n* = 202), which acts as a matched control for oral health parameters, OLK exhibited increased *S. infantis* , *Leptotrichia* spp., *Bergeyella* spp., *Porphyromonas* spp. and *F. nucleatum* . Machine learning with clinical and microbiome data could discriminate high‐risk dysplasia (moderate to severe) from low‐risk dysplasia (none or mild) (sensitivity 87.4%; specificity 76.5%). Follow‐up swabs were recovered from 58 patients, eight of whom progressed to a higher grade of dysplasia or OSCC and these eight patients exhibited a higher abundance of *Fusobacterium* species at their initial presentation.

**Conclusions:**

Our study suggests that the OLK microbiome has potential to be an aid to the prediction of dysplasia grade and the risk of malignant transformation.

**Abbreviations**
CLN: contralateral normal
HROLK: high‐risk OLK
LROLK: low‐risk OLK
OED: oral epithelial dysplasia
OLK: oral leukoplakia
OPMD: oral potentially malignant disorder
OSCC: oral squamous cell carcinoma
OTU: operating taxonomical unit

## 1 Introduction

OSCC is the most common oral cavity cancer diagnosed worldwide (National Institutes of Health 2024). As in other human cancers, oral cancer can be preceded by a precursor lesion or condition. In the mouth, these are termed oral potentially malignant disorders (OPMD), a group of mucosal conditions associated with a statistically increased risk of transformation to cancer. The global prevalence of OPMD is estimated at 4.5%, the most common in the Western World being oral leukoplakia (OLK) with an estimated global prevalence of 4.1% (Mello et al. 2018) and an overall risk of malignant transformation of 9.8% (annual rate 1.56%) (Aguirre‐Urizar, de Mendoza, and Warnakulasuriya 2021). Risk factors for the development of OLK are similar to those for oral cancer and include tobacco smoking and consumption of alcohol (Morse et al. 1996; Hashibe et al. 2000; Maserejian et al. 2006; Gueiros et al. 2018; Mello et al. 2018; Warnakulasuriya 2018). The diagnosis of OLK is made by clinical and histopathological examination, with the histology typically showing some degree of dysplasia (cytological and/or architectural atypia), which is graded as mild, moderate or severe (El‐Naggar et al. 2017), although some can show no dysplasia. Currently, the most accurate predictor of malignant transformation is the degree of dysplasia on biopsy (Schepman et al. 1998; Warnakulasuriya et al. 2011; Liu et al. 2012; Iocca et al. 2020; Aguirre‐Urizar, de Mendoza, and Warnakulasuriya 2021; Guan et al. 2023), with OLK showing severe dysplasia having a transformation rate of 24%–26% over 10 years (Mehanna et al. 2009; Warnakulasuriya et al. 2011).

Over the past 25 years it has been established that the microbiome of OSCC differs significantly from that of healthy mucosa (Perera et al. 2018; Radaic et al. 2023; Shen et al. 2023). This has focussed attention on the possible role of the microbiome in the development of OSCC resulting in several studies exploring the microbiome of OPMDs (Amer et al. 2017; Wright et al. 2023). Most studies investigating the microbiome of OPMDs conducted to date have had relatively small sample sizes. In addition, a recent systematic review by Shen et al. (2023) that included 10 microbiome studies of oral epithelial dysplasia (OED) noted significant heterogeneity with regards to sampling methods, control groups and presence of confounding factors, making it difficult to draw an overall conclusion on the microbiome of OED (Schmidt et al. 2014; Amer et al. 2017; Lee et al. 2017; Mok et al. 2017; Decsi et al. 2018; Ganly et al. 2019; Chen et al. 2021; Gopinath et al. 2021; Herreros‐Pomares et al. 2021; Heng et al. 2022). The most common findings in these studies were significant increases in the phylum Bacteroidetes and the genus *Fusobacterium* and loss of *Streptococcus* species in OED compared with healthy controls. While there is no conclusive evidence to show that microbiome changes in OLK patients may cause or drive the progression of dysplasia, it has been speculated that production of carcinogens such as acetaldehyde or a shift towards inflammatory, Gram‐negative bacteria could play a contributory role.

The current study aimed to analyse the oral microbiome in a large cohort of patients with OLK to determine if OLK sites had an altered microbiome and to determine if these microbiome changes could be used to discriminate the degree of dysplasia and therefore the risk of malignant progression. We designed four study components to investigate this:

1. A case‐control study to compare the oral microbiome of OLK patients and a healthy patient cohort to determine if poor oral health and smoking impact on the oral microbiome in OLK patients.
2. A second case‐control study that compared the specific microbiome of the OLK to matched contralateral normal sites (CLN) in the same patients. The contralateral site was used as a control as it is matched to the OLK for age, gender, oral health and smoking. This comparison allows us to identify OLK‐specific biomarkers independent of oral health parameters.
3. An analysis of the microbiome of the OLK samples only, stratified by dysplasia grade, to determine if machine learning techniques could be used to distinguish between different degrees of dysplasia.
4. A longitudinal analysis of OLKs that exhibited changes in dysplasia grade during the study to determine if any microbial species could be biomarkers of dysplastic change.

Our findings suggest that OLK patients have a specific microbiome dysbiosis and that the OLK microbiome could potentially be used to discriminate sites at lower or higher risk of malignant transformation.

## 2 Materials and Methods

### 2.1 Patients and Sampling

Ethical approval was granted by the Joint Hospitals' Research Ethics Committee (JREC Ref: 2017–11‐Chairman's actions). Following specific consent, oral mucosal swabs were collected from patients with OLK attending the Oral Mucosal Dysplasia clinics in the Dublin Dental University Hospital (DDUH) using BD BBL Culture Swab EZ (Copan Italia SpA, Brescia, Italy). Patients were swabbed at the lesion site(s) and at a CLN site if available. If the contralateral site was clinically involved, another similarly keratinised or non‐keratinised site was selected as the control site. Data on patient demographics, lesional clinical features, smoking status, alcohol consumption (units/week), denture wear, missing teeth (Hashim et al. 2016) and oral hygiene practices (toothbrushing frequency and mouthwash use) were recorded (Table S1). The presence and degree of dysplasia were established by histopathological analysis by consultant Oral and Maxillofacial Pathologists in St James's Hospital, Dublin and was graded according to the three‐tier WHO 2017 classification (mild, moderate, severe dysplasia) (El‐Naggar et al. 2017). Oral hygiene was measured by calibrated examiners using the Greene and Vermillion Simplified Oral Hygiene Index (OHI‐S, Table S1) (Greene and Vermillion 1964). Patients with diabetes mellitus, Crohn's disease, ulcerative colitis, previous gastrointestinal tract malignancy, dry mouth, or current upper respiratory tract infection were excluded, along with those who within the previous two months had taken systemic antibiotics or immunosuppressants, including corticosteroids, or who had used topical corticosteroids intra‐orally. Patients were also excluded if there was no available histopathological report related to the OLK. Healthy controls were sourced from periodontal and oral hygiene clinics in the DDUH and were subject to the same exclusion criteria. After obtaining specific consent, control subjects were examined by a clinician to ensure they had no visible mucosal abnormality and swabs were then collected from the buccal mucosa, lateral border of tongue, floor of mouth and soft and hard palate. All swabs were transported to the laboratory within 1 h and resuspended in 500 μL of TSE buffer (10 mM Tris–HCL [pH 7.8], 1 mM EDTA, 100 mM NaCl) in a screw‐top tube. They were then stored at −80°C until DNA extraction was carried out using the MasterPure DNA/RNA Purification Kit (Epicentre Biotechnologies, Madison, WI, United States) as described by Amer et al. (2017).

### 2.2 DNA Sequencing

Amplification of the V1‐V3 region of the 16S rRNA gene was carried out using the primers 27F‐YM and 519 R (27F‐YM: 5′AGAGTTTGATYMTGGCTCAG; 519 R: 5′GWATTACCGCGGCKGCTG) (Frank et al. 2008; Wade and Prosdocimi 2020). Amplification, library production and sequencing were carried out by Integrated Microbiome Resources (IMR; Dalhousie, Halifax, Canada) according to their standardised in‐house protocols described by Comeau, Douglas, and Langille (2016) and detailed here https://www.protocols.io/workspaces/integrated‐microbiome‐resource‐imr/publications. Paired‐end sequencing was performed using the Illumina 600 cycle MiSeq V3 kit to generate 300 bp paired end reads. All data are available from the NCBI (https://www.ncbi.nlm.nih.gov/bioproject/PRJNA1139804).

### 2.3 Data Analysis

The clinical and patient demographic data were analysed in RStudio using the R packages rcmdr (Fox 2016), epiR (Stevenson et al. 2023), car (Fox et al. 2023) and nnet (Ripley and Venebles 2016).

### 2.4 Microbiome Analysis

Dada2 was used to filter and process 16S reads with the following parameters: maxN = 0, maxEE = c(2,5) and truncQ = 2 (Callahan et al. 2016). Forward and reverse reads were trimmed to 290 and 270 bp, respectively and merged before chimera removal with the removeBimeraDenovo command. Following processing, a total of 548 samples yielded > 5000 reads. Taxonomy was assigned using the expanded Human Oral Microbiome Database (eHOMD) classifications (eHOMD 16S rRNA RefSeq version 15.1) (Escapa et al. 2018). Microbiome alpha diversity and community structure (beta diversity) were analysed using the packages Phyloseq (McMurdie and Holmes 2013) and MicrobiotaProcess (Xu et al. 2022).

Multivariate analysis was conducted with MaAsLin2 (Mallick et al. 2021) using relative abundance data and the default linear models (LM) setting with log transformation of the data. DESeq2 (Love, Huber, and Anders 2014) was also used to further validate any changes in species abundance. For predictive modelling, microbial features of interest were identified by random forest (RF) analysis using SIAMCAT following log transformation of the data and z‐score standardisation. The z‐scores of the top 50 microbial features identified in SIAMCAT were used for additional predictive modelling analysis both with and without clinical and patient factors using the R packages rpart (Therneau, Atkinson, and Ripley 2023) and randomForest (Liaw and Wiener 2002). Up to 10,000 iterations of Classification trees using random subsets of 90% of cases were carried out to estimate sensitivity, specificity, positive predictive value (PPV), negative predictive value (NPV) and classification accuracy with corresponding confidence intervals (CI), along with ROC curves and Gini indices (measures of the impurity of the dataset). RFs were then run with 500 trees with 25 (for microbial data) or 30 (for combined microbial and clinical/patient data) random variables for each tree.

## 3 Results

**FIGURE 1:**
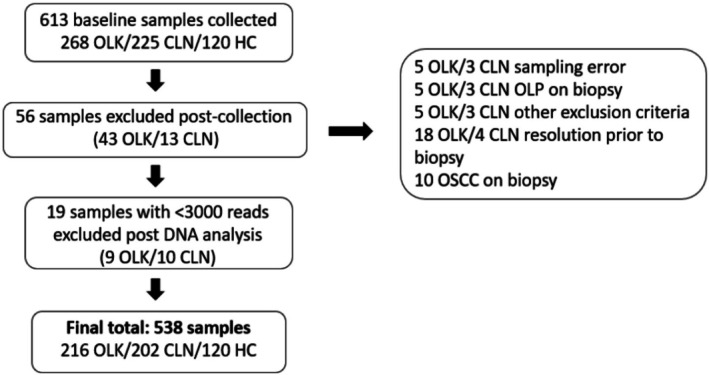
An outline of sample processing. From the initial 613 samples collected, 56 samples were removed due to errors, diagnosis as oral lichen planus (OLP) or other exclusion criteria (see methods). Following DNA sequence processing, 538 samples were available for analysis including 216 OLK swabs and 202 CLN site swabs from patients and 120 swabs from 61 healthy subjects.

**FIGURE 2:**
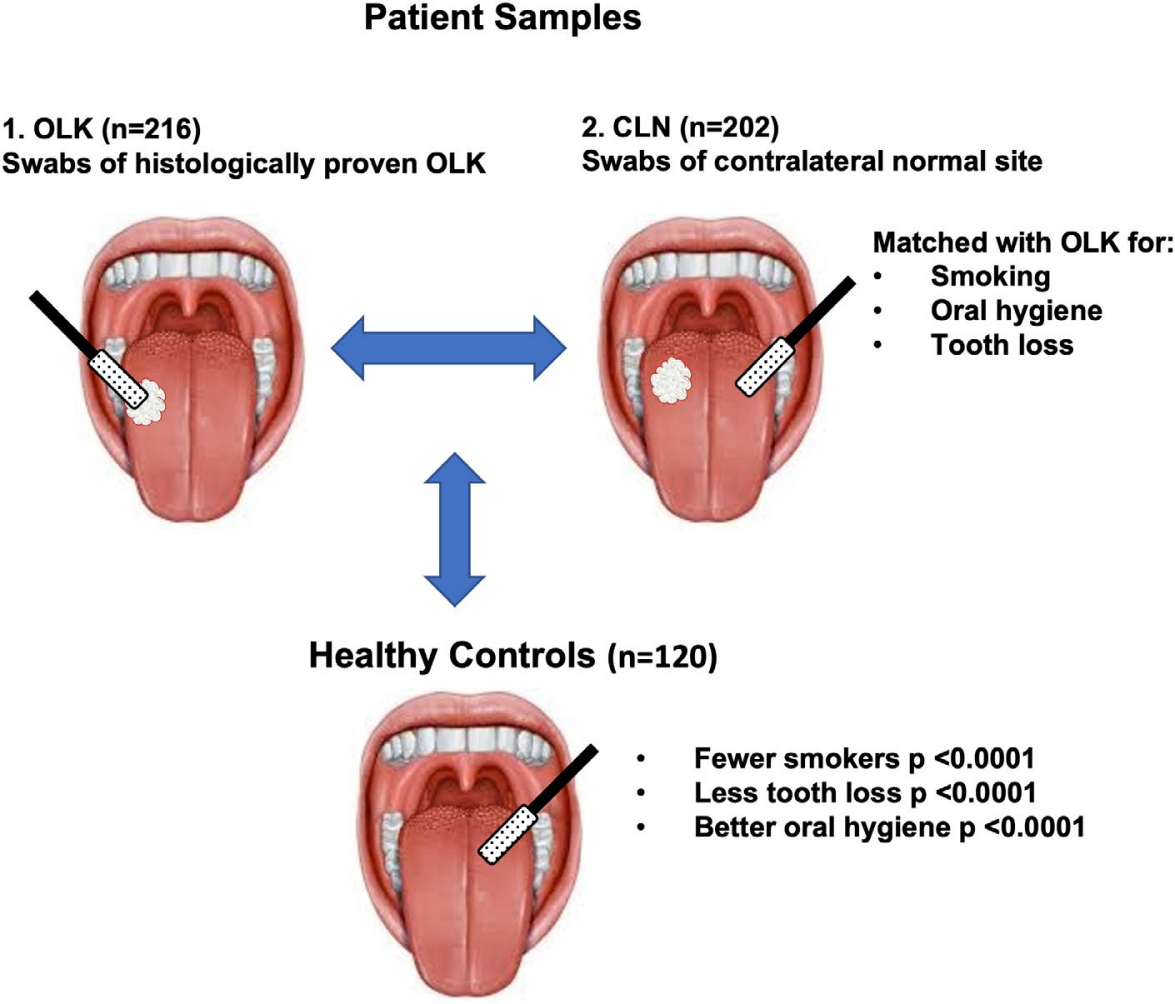
An outline of study design. Patients were sampled by mucosal swabbing at disease sites (i.e., histologically proven OLK) and at contralateral normal (CLN) mucosal sites to match for variables such as smoking, oral hygiene and tooth loss. We also recruited a cohort of healthy controls with better oral health to investigate the impact of these factors on the mucosal microbiome (See Table S1 for detailed comparison).

A total of 216 OLK swabs and 202 CLN site swabs from 177 patients were available for analysis following exclusions and data processing (Figure 1 and Table 1). The average patient age was 58.3 years (median 58 years) and present or past tobacco use was reported by 80.8% (*n* = 143) of patients (Table S1). The majority (83.8%) of the OLKs were homogeneous, 35 were non‐homogeneous, of which seven were ulcerated (Table 1). All degrees of dysplasia were represented (Table 1) and 10 suspected leukoplakias were diagnosed as OSCC following biopsy (Table 1). A healthy control group of patients without OLK attending periodontal and oral hygiene clinics at the Dental Hospital was also recruited (*n* = 120 swabs from 61 patients; Table 1). These patients were generally younger, smoked less, were more dentate and had better oral hygiene compared to the OLK cohort (Figure 2 and Table S1).

**TABLE 1:** Details of swabs recovered from patients (OLK and CLN sites) and healthy controls including clinical appearance and degree of dysplasia of OLKs.

Sample data	% OLK^a^ (*n* = 216)	% CLN^b^ (*n* = 202)	% HC^c^ (*n* = 120)
Sites samples (*n* = 428)			
Floor of mouth/ventral tongue	28.2 (61)	12.9 (26)	14.2 (17)
Buccal mucosa/Alveolus/Gingiva	37 (80)	41.6 (84)	35.9 (43)
Dorsum/lateral tongue	22.7 (49)	36.1 (73)	35.9 (43)
Palate	12 (26)	9.4 (19)	14.2 (17)
Clinical appearance (*n* = 216)			
Homogeneous	83.8 (181)	NA	NA
Non‐homogeneous of which ulcerated	16.2 (35) 3.2 of total (7)	NA	NA
Histological findings (*n* = 226)			
No dysplasia	4.9 (11)	NA	NA
Mild dysplasia	35.4 (80)	NA	NA
Moderate dysplasia	33.6 (76)	NA	NA
Severe dysplasia	21.7 (49)	NA	NA
OSCC	4.4 (10)	NA	NA
Binary categorisation of dysplasia (*n* = 226)			
Low‐risk OLK (no/mild dysplasia)	40.3 (91)	NA	NA
High‐risk OLK (moderate/severe dysplasia)	55.3 (125)	NA	NA
OSCC	4.4 (10)	NA	NA

^a^ Oral leukoplakia.

^b^ Contralateral normal mucosa.

^c^ Healthy control patient.

### 3.1 Microbiome of OLK Patients Compared to Healthy Cohort

**FIGURE 3:**
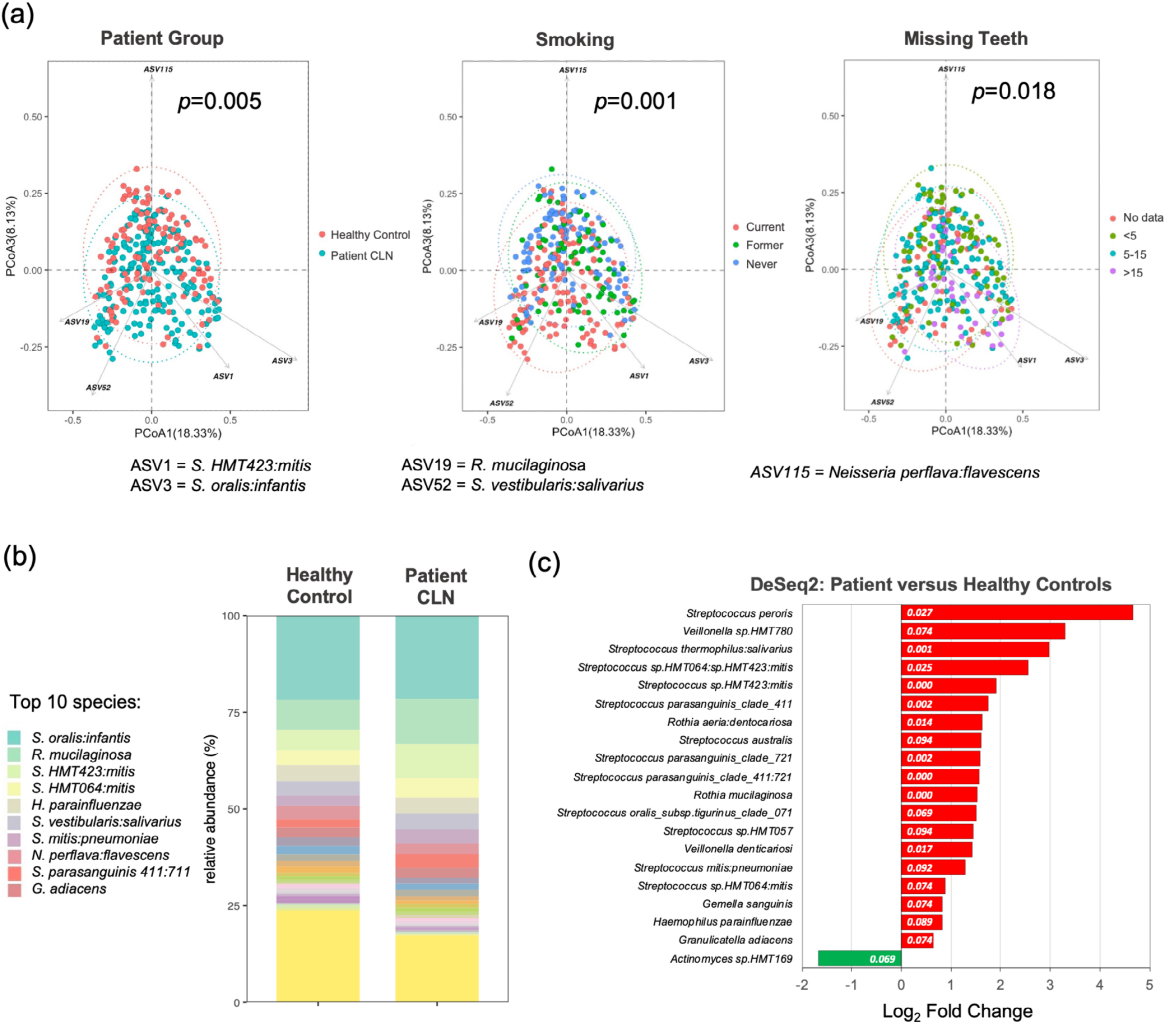
Analysis of oral microbiome community structure in OLK patients and healthy controls. (a) PCoA of Bray–Curtis dissimilarity values showing significant divergence in community structure (PERMANOVA *p* values) in OLK patients and healthy controls, smokers and in different categories of tooth loss. (b) Bar plot showing mean relative abundance of species in OLK patients and controls. (c) Statistically significant changes in species relative abundance in OLK patients versus healthy controls were identified using DeSeq2. Significance (*q* values) is shown in white.

Two case‐control analyses were performed in this study (Figure 2). The first analysis aimed to determine how the oral environment of the OLK patient, including factors such as smoking, tooth number and oral hygiene (Table S1), influenced oral microbiome composition. To determine the impact of these factors, we compared patient microbiomes to a healthy control group (*n* = 120 swabs from 61 patients; Figure 2) who were generally younger, were less likely to smoke, were more dentate and had better oral hygiene compared to the OLK cohort (Table S1). In this analysis, we compared the contralateral normal (CLN) sites in OLK patients to the healthy patient cohort. Measures of microbiome alpha diversity, which compare sample richness in terms of numbers of taxa and the evenness of their distribution, were similar in both cohorts with a modest increase in evenness (Pielou) and biodiversity (Simpson) in the healthy populations (Figure S1). Comparison of microbiome composition (beta diversity) by PCoA showed that microbiome populations from OLK patients were significantly different in composition from healthy controls (PERMANOVA *p* = 0.005; Figure 3a), with no significant difference in dispersion (PERMDISP *p* = 0.11). This change in structure was associated with reduced *Neisseria* sp. and increased *S. mitis, S. oralis, S. salivarius* and *Rothia mucilaginosa* (Figure 3a,b). Analysis of both OLK patients and controls showed that current smoking and high levels of tooth loss (> 15 missing teeth, indicative of periodontal disease) were the main factors influencing microbiome composition in OLK patients (Figure 3a). Increased abundances of several *Streptococcus* spp., *Veillonella* spp., *R. mucilaginosa* and *R. aeria:dentocariosa* in OLK patients compared to healthy controls were statistically significant (Figure 3c and Table S2). However multivariate analysis using MaAsLin2 showed that increased abundance of *S. parasanguinis* , *S. mitis* and *S. salivarius* in OLK patients was not significant when the data were adjusted for smoking and tooth loss (Figure S2).

### 3.2 Microbiome of OLK Compared to Contralateral Healthy Sites

**FIGURE 4:**
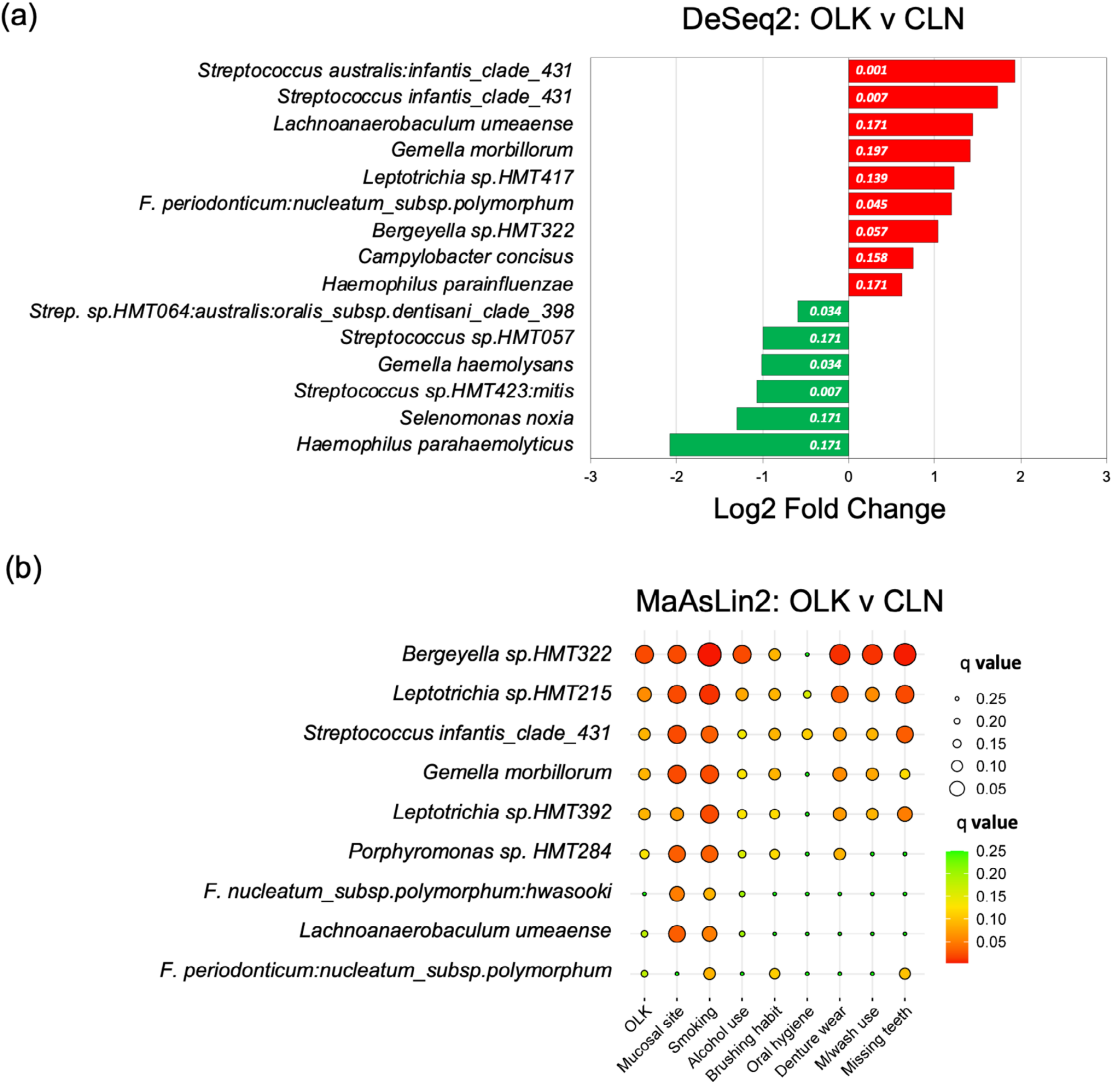
(a) Statistically significant changes in species relative abundance between OLK and CLN sites in patients identified using DeSeq2. Significance (*q* values) is shown in white. (b) Results of multivariate analysis using MaAsLin2 to identify species with increased abundance on OLK versus CLN sites in patients. Symbols indicate significance (*q* values) following adjustment for mucosal site, smoking, alcohol use, brushing frequency, oral hygiene levels, denture wear, mouthwash use and number of missing teeth (see Table S1 for categories).

The initial comparison of the OLK patients to the healthy cohort identified microbiome changes in patients linked to smoking and tooth loss. Next, a second case‐control study was carried out, controlling for oral health parameters and identifying OLK‐specific biomarkers, independent of smoking and tooth loss. To do this, we compared the microbiome of the OLK to CLN sites. As CLN swabs were recovered from the same patients we consider them matched controls for the influence of smoking, oral hygiene and periodontal disease on the microbiome (Figure 2). We did not observe any major changes in microbiome community structure between OLK and CLN samples in terms of alpha diversity (Figure S1) or beta diversity (Figure S3). However, we could identify specific taxa that were significantly more abundant on OLK compared to CLN sites, the most significant including *S. infantis* clade 431, *Leptotrichia* sp. HMT215, *Bergeyella* sp. HMT322, *Gemella morbillorum* , *Porphyromonas sp*. HMT284 and *F. nucleatum* subsp. *polymorphum* (Figure 4). We carried out an extensive multivariate analysis that showed that most of these taxa showed increased abundance when data were adjusted for oral site, smoking, missing teeth, age and gender (Figure 4b). However, the significance of these increases was reduced when oral hygiene and brushing frequency were taken into account (Figure 4b).

### 3.3 The Degree of Epithelial Dysplasia Influences the Microbiome

**FIGURE 5:**
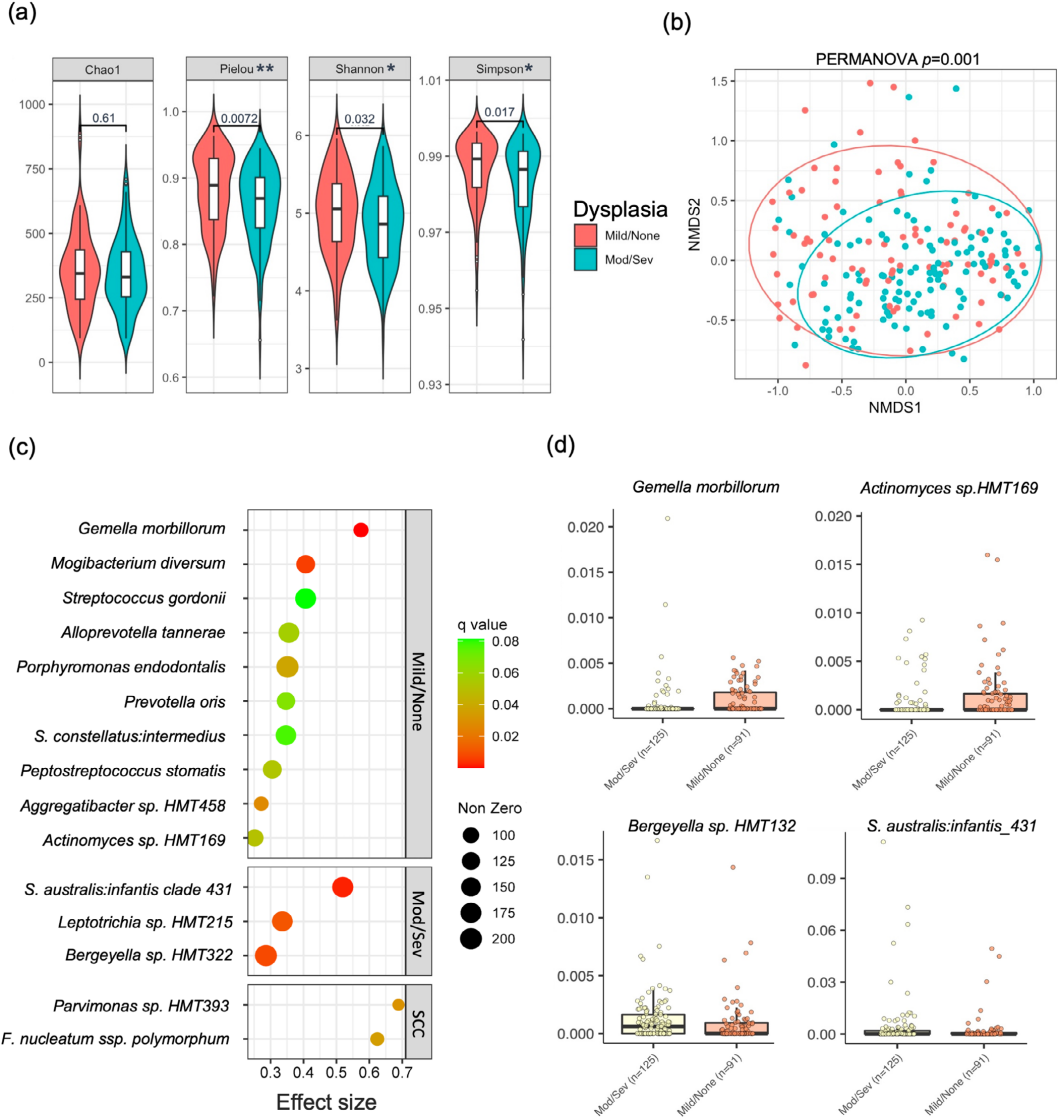
Comparison of the microbiome of OLK with no/mild dysplasia (low‐risk OLK) and moderate/severe dysplasia (high‐risk OLK). (a) Alpha diversity metrics of no/mild dysplasia (red) and moderate/severe dysplasia (blue) OLK. Significance indicated by *p* values of Wilcoxon signed‐rank tests. (b) Comparison of microbiome community structure of no/mild dysplasia (red) and moderate/severe dysplasia OLK (blue) using non‐metric multi‐dimensional scaling (NMDS) of Bray–Curtis dissimilarity values. (c) Results of MaAsLin2 analysis to identify species significantly associated with no/mild or moderate/severe dysplasia. Symbol colour indicates level of significance (*q* values) and size equals number of non‐zero values. (d) Box plots showing relative abundances of selected species in (c).

We next focused exclusively on the OLK samples to investigate if the microbiome changed with increasing degree of dysplasia. For this analysis, we classified dysplasia in a binary manner as low‐risk (i.e., no/mild dysplasia; LROLK, *n* = 91) or high‐risk (i.e., moderate/severe dysplasia; HROLK, *n* = 125) (Küffer and Lombardi 2002; Kujan et al. 2006; Speight et al. 2015; Reibel et al. 2017; Odell et al. 2021). We also included the ten OSCC swabs as comparators. Analysis of alpha diversity showed that HROLK exhibited significantly decreased evenness and biodiversity compared to LROLK (Figure 5a). Analysis of beta diversity using Bray‐Curtis values also showed that HROLK had a diverging population structure from LROLK (Figure 5b; PERMANOVA *p* = 0.001) but no significant difference in dispersion (PERMDISP *p* = 0.7).

HROLK exhibited a number of species enrichments, including *Bergeyella* sp. HMT322, *Leptotrichia* sp. HMT215 and *S. australis:infantis* clade 431 (Figure 5c). LROLK exhibited an increased abundance of *Gemella morbillorum* , *Mogibacterium diversum* and *Porphyromonas endodontalis* (Figure 5c). OSCC exhibited higher abundance of *F. nucleatum* subsp. *polymorphum* and *Parvimonas* sp. HMT393 (Figure 5c). Similar differences were identified when OLKs were classified using the 3‐tier WHO 2017 classification, with *F. nucleatum* also associated with severe dysplasia (Figure S4). In multivariate analysis, these differences were independent of smoking, oral site and tooth loss, although significance was reduced when the data were adjusted for oral hygiene (Figure S5).

### 3.4 Predictive Modelling With Microbiome Data

Machine learning with randomForest (RF) (Liaw and Wiener 2002) was next applied to the data to determine if clinical and microbiome information could discriminate LROLK from HROLK. Using the patients' clinical and demographic information only (Table S1), we could discriminate HROLK from LROLK with a sensitivity of 76.3% and specificity of 66.1% (Table S3). The AUC value, defined as the area under the receiver operating characteristic (ROC) curve, was 78.5% (Figure S6a) and, based on the illustrative Classification tree (Figure S6b), a non‐homogeneous clinical appearance was almost always associated with HROLK (node 13). Using microbiome data, we could discriminate HROLK from LROLK with a sensitivity of 85.5% and a specificity of 76.5% (Table S3). The AUC value, as defined using the ROC, was 78.8% (Figure S7). LROLK could be predicted by an increased abundance of several taxa including *Mogibacterium diversum, Prevotella oris, Porphyromonas endodontalis* and *Gemella morbillorum*.

**FIGURE 6:**
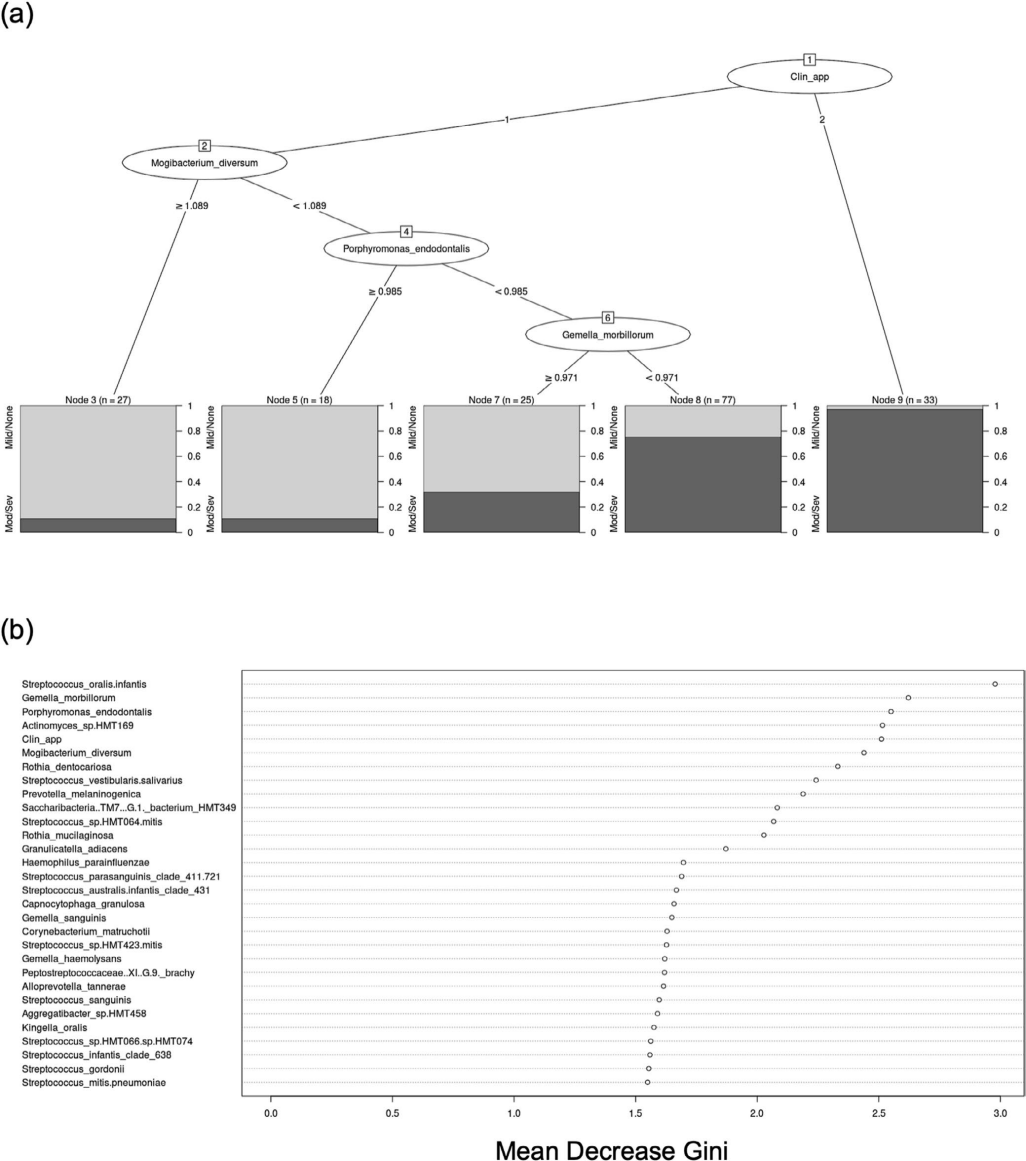
(a) Graphic of a Classification tree from RF modelling using the z‐scores of the top 50 microbial features from initial SIAMCAT analysis and clinical and patient factors. (b) Graphic showing the Gini index of the most discriminatory features found in the analysis.

Finally, predictive modelling was carried out using the most discriminatory clinical/patient data (shown in Figure S6) combined with microbial abundance data. Again, non‐homogeneous clinical appearance was almost always associated with HROLK (Figure 6a). If the OLK was homogeneous, increased abundance of the microbial features *M. diversum, Por. endodontalis* and *G. morbillorum* could be used to identify LROLK. The Gini index in Figure 6b shows the most discriminatory features in this analysis were *S. oralis:infantis*, *G. morbillorum, Por. endodontalis, Actinomyces* sp. HMT169, clinical appearance of the OLK and *M. diversum*. These combined features could differentiate LROLK from HROLK with a sensitivity of 87.4% and specificity of 76.5% (Table S3). The AUC of the ROC was 88% (Figure S8). Interestingly and as for the microbiome modelling, the error rate was much lower for predicting HROLK (20.5%) than for LROLK (35.6%).

### 3.5 Longitudinal Analysis of the OLK Microbiome

**FIGURE 7:**
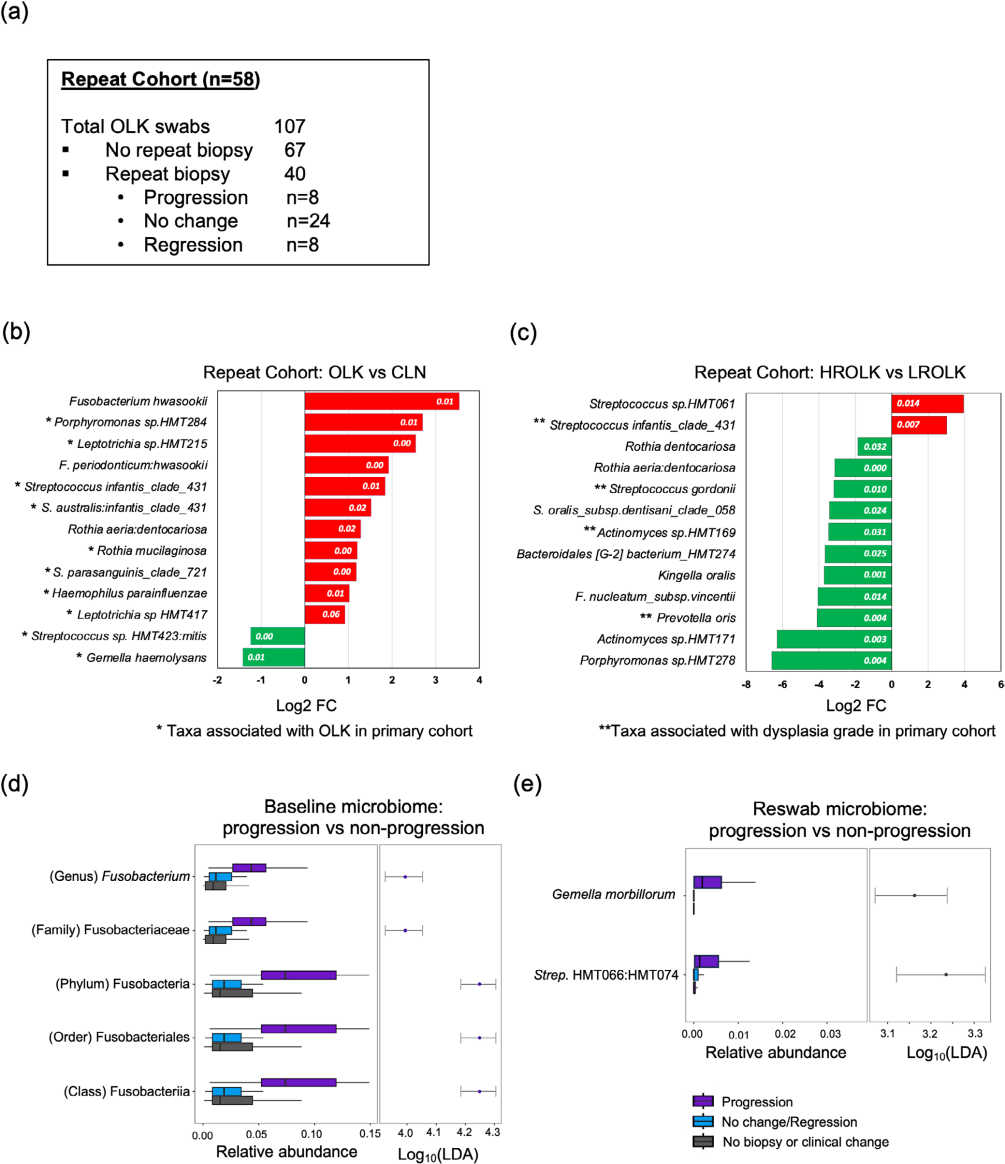
(a) Characteristics of the repeat visit patient cohort. (b) Statistically significant changes in species relative abundance between OLK and CLN and (c) HROLK and LROLK sites in repeat cohort patients identified using DeSeq2. Adjusted *p* values are shown in white (d) Comparison of the baseline microbiomes of patients with repeat histopathology including those who exhibited an increase in dysplasia (progression) or no change/reduced dysplasia (No change/regression). (e) Comparison of the repeat sample microbiomes of patients with repeat histopathology including those who exhibited an increase in dysplasia (progression) or no change/reduced dysplasia (No change/regression).

As part of a longitudinal analysis, follow‐up swabs (*n* = 107) were recovered from 58 patients twelve months after the initial swab (Figure 7a). Upon re‐examination, 40 white patches exhibited clinical change and were re‐biopsied, with 24 showing no change in the degree of dysplasia from the previous biopsy, 8 showing regression of dysplasia and 8 showing progression (Figure 7a). Compared to the main cohort, the repeat visit cohort had fewer mild and no dysplasia samples (22.3% compared to 42%) and was dominated by OLK with moderate dysplasia (59.2% compared to 35%; Table S4). Comparison of the microbiomes of OLK and CLN mucosa in the repeat cohort largely confirmed the OLK‐microbiome identified in the primary cohort (Figure 7b). Binary risk classification of the repeat cohort into LROLK and HROLK confirmed the association of *S. infantis* clade 431 with HROLK and *Actinomyces* spp. HMT169, *Prevotella oris* and *S. gordonii* with LROLK (Figure 7c).

We next examined if the microbiome had any correlation with a change in degree of dysplasia in those repeat cohort samples that were re‐biopsied (*n* = 40) (Figure 7a). We first examined the baseline microbiomes of samples with repeat histopathology and found that those OLKs that exhibited progression in the degree of dysplasia (*n* = 8) had significantly higher levels of Fusobacteria (including the genus *Fusobacterium*) at baseline compared to those that did not exhibit progression (*n* = 32) or were not biopsied (*n* = 67) (Figure 7d). Upon re‐swab, the OLKs that progressed exhibited higher levels of *Gemella morbillorum* and *Streptococus* sp. HMT066:HMT074 compared to those that did not progress (Figure 7e). We also examined temporal changes in the microbiome from baseline to repeat swab. The OLKs that exhibited progression in the degree of dysplasia (*n* = 8) showed a significant reduction in the level of *Mogibacterium* spp. over time and those OLKs that regressed or showed no change in degree of dysplasia (*n* = 32) showed increased levels of *Leptotrichia* spp. and *Actinomyces* spp. when re‐examined (Figure S9).

## 4 Discussion

In this study, we present the most comprehensive analysis of the microbiome in patients with OLK published to date. We used a study design similar to our previously published pilot study where we used CLN mucosa from the OLK patients as a matched control to identify OLK‐specific biomarkers (Amer et al. 2017). We also compared the oral microbiome of OLK patients to that of a cohort of healthy control patients as our earlier work had highlighted the significant impact of lifestyle and oral health on the oral microbiome (Galvin et al. 2023). In general, the OLK patients recruited in this study smoked more, had poorer oral hygiene, and had lost more teeth (likely due to periodontal disease) compared to the healthy control cohort (Table S1). Comparison of this healthy control patient microbiome to the microbiome of the OLK patients identified specific shifts in patient microbiome structure that appear to be linked to smoking and tooth loss, characterised by increased abundance of species including *R. mucilaginosa, S. thermophilus:salivarius* and several OTUs of *S. parasanguinis*. This shift in community structure is very similar to the microbiome associated with high acetaldehyde generation described by Yokoyama et al. (2018). Acetaldehyde is a carcinogen and is the first metabolite of alcohol (Amer et al. 2020). The discovery of this community type in OLK patients raises the question as to whether acetaldehyde generation, either *de novo*, or following alcohol consumption could contribute to the aetiology of OLK or contribute to malignant progression. Studies of saliva from oral cancer patients and smokers have shown that their saliva has the capacity to generate higher levels of acetaldehyde in vitro compared to healthy non‐smokers (Homann et al. 2000, 2001; Marttila et al. 2013). Our current data suggest that smoking could induce shifts in the microbiome towards a population that generates acetaldehyde from alcohol, which could be a contributory factor in the synergy of alcohol and smoking in the aetiology of oral cancer. Establishing this will require specific investigations to determine cause and effect, which were beyond the remit of the current study.

The subsequent comparison of the OLK microbiome to the matched control CLN mucosa identified OLK‐specific biomarkers. This analysis validated our previous pilot study (Amer et al. 2017) and also identified additional taxa including *S. infantis* clade 431 that show consistently increased abundance on OLK. However, the multivariate analysis shows that some of these associations are less significant when oral hygiene and tooth brushing frequency are included. We speculate that good oral hygiene practices may reduce the microbial biomass on the OLK making it more difficult to detect significant increases in species abundance. The abundance of some periodontal pathogens on OLK was strongly associated with periodontal diseases (indicated by missing teeth), as for example the association of *Porphyromonas* sp. HMT284 and some *Fusobacterium nucleatum* strains was less significant when the data were corrected for a number of missing teeth.

Interestingly, we found that the degree of dysplasia influenced the microbiome of the OLK and several dysplasia‐specific biomarkers could be identified, even when the analysis is corrected for oral health parameters and missing teeth. This suggests that changes in epithelial biology or topology that occur with increasing dysplasia influence the structure of the adherent microbiome, which could be diagnostically useful. Our multivariate analysis showed that these changes were independent of smoking and oral site. Some taxa were influenced by oral hygiene, however *S. australis:infantis* clade 431 (*p*adj = 0.059) and *Bergeyella* sp. HMT322 (*p*adj = 0.051) was still associated with severe dysplasia even after adjustment for oral hygiene, and *G. morbillorum* (*p*adj = 0.059) was still associated with mild dysplasia after adjustment for oral hygiene. These findings prompted us to investigate whether machine learning techniques could take these microbiome data and, in conjunction with clinical parameters, develop a predictive tool to determine whether an OLK is in the LROLK or HROLK category. Combined clinical and microbiome data discriminated LROLK from HROLK with a sensitivity of 87.4% and specificity of 76.5%. In conjunction with non‐homogeneous clinical appearance, which identified many HROLKs, risk could be further discriminated by the levels of several taxa associated with LROLK such as *M. diverersum, Prev. oris, Por. endodontalis* and *G. morbillorum* .

Finally, we carried out a follow‐up analysis on repeat swabs from a cohort of 58 patients. Comparison of the OLK and CLN sites in these patients confirmed the presence of the OLK‐specific microbiome. Several of the OLKs re‐examined in the repeat cohort exhibited increased levels of dysplasia following repeat histopathological analysis (*n* = 8). Although the numbers are small, baseline samples taken from these patients who later progressed harboured significantly higher levels of *Fusobacterium* spp. compared to samples that exhibited no change or regressed. *Fusobacterium* spp. have been linked to malignant progression of cancers in the gastrointestinal tract (Rubinstein et al. 2019; Chen et al. 2020) and our finding warrants further investigation to determine if levels of this bacterium influence the progression of dysplasia in OPMDs.

Unfortunately, this study cannot fully elucidate cause and effect relationships between the microbiome and progression of dysplasia. This would require a substantial longitudinal analysis, beyond the preliminary longitudinal analysis shown in Figure 7. Detailed mechanistic analysis of the interaction of the microbiome and the tissue may require microbiome analysis of biopsy samples. As our study focused on the diagnostic potential of the microbiome, our analysis used a non‐invasive sampling method and was not designed to sample within the tissue. Our study was limited to bacteriome analysis and future studies should also investigate the presence of fungi and viruses and the influence of smoking load on the entire microbiome.

## 5 Conclusion

This study for the first time comprehensively investigates the oral microbiome of a cohort of OLK patients and identifies a pattern of dysbiosis similar to that identified in individuals that generate high levels of acetaldehyde in saliva. We also demonstrate that microbiome data may be a useful adjunct to clinical information in predicting the degree of dysplasia and risk of malignant progression. Future investigations of the microbiome of OLK should assess whether metagenomic sequencing could increase the reproducibility and discriminatory power of these analyses.

## Author Contributions

**Sheila Galvin:** investigation, data curation, formal analysis, writing‐original draft. **Bahman Honari:** investigation, formal analysis. **Sviatlana Anishchuk:** project administration, investigation, data curation. **Claire M. Healy:** conceptualization, funding acquisition, project administration, supervision, writing – review and editing. **Gary P. Moran:** conceptualization, funding acquisition, project administration, supervision, formal analysis, writing – review and editing.

## Ethics Statement

Oral mucosal swabs were collected from patients with OLK attending the Oral Mucosal Dysplasia clinics in the Dublin Dental University Hospital with specific, informed and signed consent. Ethical approval was granted by the Joint Hospitals' Research Ethics Committee (JREC Ref: 2017–11‐Chairman's actions).

## Conflicts of Interest

The authors declare no conflicts of interest.

## Supporting information


Data S1.

## Data Availability

All sequence data and detailed patient metadata are available from the NCBI (https://www.ncbi.nlm.nih.gov/bioproject/PRJNA1139804).
