# Supplementary material for: Oral Leukoplakia Microbiome Predicts the Degree of Dysplasia and is Shaped by Smoking and Tooth Loss
Source: Oral Dis. 2025 Feb 4;31(6):1704–16. doi: 10.1111/odi.15272 (PMC12291422; doi:10.1111/odi.15272)
Supplement: Supplementary file 1 — Data S1. [file ODI-31-1704-s001.pdf]

Table S1. Comparison of OLK patient and control patient demographics and general oral health parameters

Demographic	Controls	Patients with OLK	p value[a]
Total	61	177	
**Gender**			
Males:Females	21:40	92:85	**0.003**
**Age range**			
<40	17	18	
40-60	30	83	
>60	13	76	
Not recorded	1	0	**0.0001**
**Smoking status**			
Current smoker	15	92	
Former smoker (>1 year cessation)	15	51	
Never smoker	31	34	**0.000005**
**Alcohol units/week**			
0	13	37	
1 to 10	41	93	
11 to 20	2	22	
More than 20	5	25	0.08
**Missing teeth**			
<5	26	25	
5 to 15	26	75	
More than 15	7	34	
Not recorded	2	43	**0.0005**
**OHI Score[b]**			
Good (OHI-S < 0.5)	37	40	
Fair (OHI-S 0.5-1)	15	60	
Poor (OHI-S > 1)	7	37	
Not recorded	2	40	**0.00002**
**Brushing times/day**			
<1	0	18	
1	5	28	
>1	56	120	
Not recorded	0	11	**0.002**
**Denture**			
Complete	1	7	
Partial	7	32	
None	53	138	0.22
**Mouthwash use**			
No	26	83	
Yes	35	94	0.1

[a] Association testing carried out using Pearson's Chi-squared, Fisher's exact and Fisher's exact with simulated p values based on 2000 replicates tests as appropriate

[b] Oral Hygiene index, Greene JG, Vermillion JR (1964) J Am Dent Assoc 68: 7–13.

Fig. S1

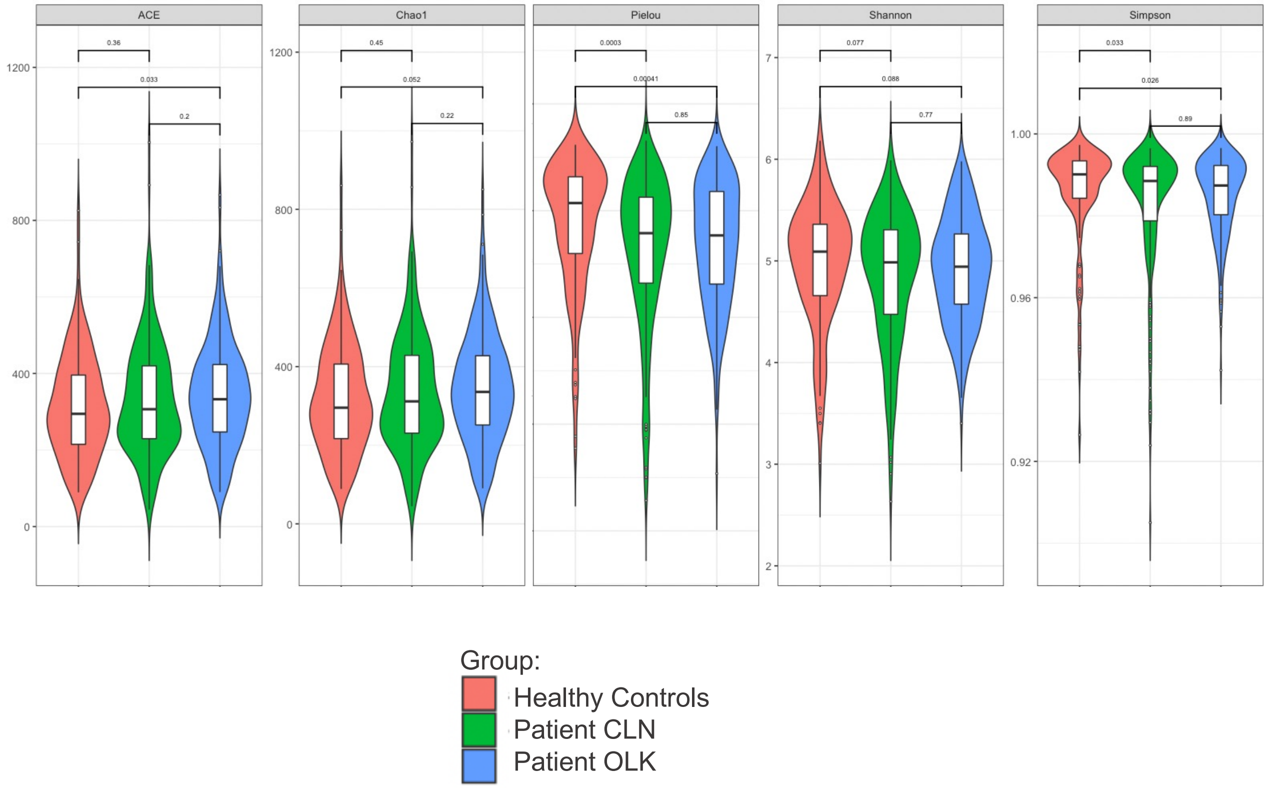

Fig. S2. Alpha diversity metrics in the healthy control patient cohort compared to the patient OLK and healthy mucosal (CLN) sites. Significance indicated by p values of Wilcoxon signed-rank tests.

Table S2. Significant changes in species relative abundance in OLK patients versus healthy controls identified using MaAsLin2. Species with greater abundance in OLK patients are highlighted in orange, those with higher abundance in healthy controls are highlighted in green.

Species	MaAsLin2 coefficient	stderr[a]	Number[b]	N.not.0[c]	pval[d]	qval[e]
Streptococcus.mitis.pneumoniae	1.639707371	0.557272	322	203	0.003495	0.025136
Rothia.aeria.dentocariosa	1.348048147	0.377799	322	168	0.000415	0.005937
Veillonella.denticariosi	1.253045722	0.343926	322	181	0.000314	0.005869
Streptococcus.parasanguinis_clade_411.721	1.235811923	0.43983	322	263	0.005263	0.030756
Streptococcus.parasanguinis_clade_411	1.058069091	0.438292	322	181	0.016336	0.067886
Streptococcus.australis.infantis_clade_431	1.010366245	0.350426	322	96	0.004202	0.026851
Veillonella.rogosae.dispar	0.948787704	0.437258	322	209	0.030753	0.102693
Streptococcus.oralis_subsp.tigurinus_clade_070	0.938657989	0.344531	322	77	0.006795	0.035296
Streptococcus.parasanguinis_clade_721	0.863413736	0.40635	322	197	0.034369	0.112755
Streptococcus.oralis_subsp.tigurinus_clade_071	0.756483863	0.344546	322	127	0.028838	0.10175
Streptococcus.sp.HMT423.mitis	0.697127687	0.388039	322	270	0.073352	0.193194
Streptococcus.sp.HMT057	0.692022923	0.395072	322	123	0.080794	0.202423
Streptococcus.infantis_clade_431.mitis	0.680307287	0.236608	322	58	0.004308	0.026851
Streptococcus.thermophilus.salivarius	0.624836531	0.188173	322	59	0.001002	0.009864
Streptococcus.sp.HMT064.sp.HMT423.mitis	0.579942887	0.239813	322	57	0.016151	0.067886
Mogibacterium.timidum	0.531483751	0.263389	322	58	0.04444	0.131909
Mycoplasma.faucium	0.472796433	0.258119	322	50	0.067926	0.18146
Veillonella.sp.HMT780	0.355204806	0.203034	322	36	0.081166	0.202423
Porphyromonas.sp.HMT277	0.275179423	0.149795	322	39	0.067131	0.18146
Streptococcus.sp.HMT064.oralis_subsp.dentisani_c	-0.426323238	0.238309	322	46	0.074569	0.193672
Streptococcus.salivarius	-0.441560597	0.216139	322	45	0.041877	0.128377
Lachnoanaerobaculum.umeaense	-0.447215635	0.19808	322	65	0.024635	0.090328
Haemophilus.sp.HMT036	-0.487847275	0.253925	322	75	0.055591	0.157508
Treponema.denticola	-0.513111943	0.21994	322	50	0.02027	0.077355
Actinomyces.oris	-0.515309226	0.207275	322	59	0.013424	0.062759
Alloprevotella.sp.HMT914	-0.535305179	0.183265	322	37	0.003738	0.025888
Fusobacterium.nucleatum_subsp.animalis	-0.544455082	0.228319	322	56	0.017679	0.071378
Eikenella.corrodens	-0.550809638	0.262739	322	95	0.036831	0.116734
Selenomonas.artemidis	-0.55506239	0.24694	322	51	0.025272	0.090882
Prevotella.nanceiensis	-0.601606659	0.253927	322	92	0.018419	0.071759
Campylobacter.concisus	-0.603581625	0.324962	322	192	0.064174	0.176478
Actinomyces.sp.HMT180.odontolyticus	-0.626848414	0.369782	322	123	0.091013	0.215437
Cardiobacterium.hominis	-0.632779053	0.1904	322	53	0.000992	0.009864
Tannerella.sp.HMT286	-0.636047896	0.275664	322	74	0.021674	0.081062
Capnocytophaga.granulosa	-0.656148582	0.263432	322	88	0.013253	0.062759
Bacteroidales..G.2..bacterium_HMT274	-0.666796525	0.269303	322	89	0.013803	0.062955
Treponema.socranskii	-0.670562128	0.188766	322	49	0.000439	0.005937
Capnocytophaga.gingivalis	-0.67655075	0.213542	322	83	0.001682	0.014295
Streptococcus.sp.HMT066	-0.70671923	0.416842	322	164	0.090969	0.215437
Neisseria.oralis	-0.715472465	0.215176	322	43	0.000987	0.009864
Selenomonas.noxia	-0.733928341	0.37114	322	98	0.048843	0.142714
Streptococcus.oralis_subsp.oralis	-0.735273397	0.338196	322	86	0.03043	0.102693
Leptotrichia.sp.HMT215	-0.7393534	0.310767	322	117	0.01794	0.071378
Leptotrichia.hongkongensis	-0.740601311	0.255958	322	99	0.004072	0.026851
Tannerella.forsythia	-0.756146328	0.252097	322	89	0.002917	0.02182
Stomatobaculum.sp.HMT097	-0.757751766	0.245793	322	86	0.002228	0.018118
Streptococcus.oralis_subsp.dentisani_clade_398	-0.759630329	0.375987	322	117	0.044179	0.131909
Corynebacterium.matruchotii	-0.770876763	0.276779	322	141	0.005669	0.031372
Lachnospiraceae..G.3..bacterium_HMT100	-0.776512694	0.258641	322	47	0.002891	0.02182
Neisseria.elongata	-0.776883683	0.233049	322	54	0.000958	0.009864
Rothia.dentocariosa	-0.785061396	0.361217	322	255	0.030485	0.102693
Actinomyces.sp.HMT448	-0.78604978	0.244583	322	48	0.001443	0.012851
Streptococcus.gordonii	-0.803798534	0.46831	322	132	0.08706	0.214214
Campylobacter.gracilis	-0.805436592	0.323538	322	110	0.013301	0.062759
Oribacterium.sinus	-0.834641696	0.345001	322	175	0.01611	0.067886
Bergeyella.sp.HMT322	-0.859198736	0.242088	322	145	0.000444	0.005937
Streptococcus.intermedius	-0.863757491	0.259497	322	54	0.000975	0.009864
Haemophilus.sputorum	-0.870341065	0.244452	322	53	0.000427	0.005937
Gemella.morbillorum	-0.870628559	0.31907	322	59	0.006711	0.035296
Neisseria.bacilliformis	-0.874814206	0.196232	322	34	1.15E-05	0.000428
Porphyromonas.gingivalis	-0.88501579	0.431396	322	68	0.04103	0.127878
Actinomyces.sp.HMT172.sp.HMT180.odontolyticus	-0.901181816	0.323804	322	63	0.005704	0.031372
Gemella.haemolysans	-0.912749846	0.487433	322	238	0.06204	0.173158
Actinomyces.sp.HMT172	-0.998688456	0.353077	322	118	0.004971	0.029987
Prevotella.oris	-1.034448794	0.420642	322	111	0.014453	0.06435
Capnocytophaga.sputigena	-1.051633493	0.281203	322	70	0.000218	0.004534
Neisseria.perflava.flavescens	-1.138025105	0.581602	322	182	0.051251	0.147445
Haemophilus.parainfluenzae	-1.199424962	0.369929	322	278	0.001311	0.012254
Actinomyces.sp.HMT175	-1.27517277	0.296932	322	81	2.32E-05	0.000725
Kingella.oralis	-1.543325098	0.389139	322	145	9.02E-05	0.00241
Neisseria.macacae.flava.mucosa.sicca	-1.544719557	0.404907	322	130	0.000163	0.00382
Streptococcus.sanguinis	-1.599412346	0.335039	322	190	2.75E-06	0.000129
Actinomyces.sp.HMT169	-1.745789015	0.320343	322	110	1.01E-07	6.29E-06
Lautropia.mirabilis	-1.807590282	0.32197	322	117	4.29E-08	4.01E-06
Rothia.aeria	-2.050367063	0.330079	322	121	1.63E-09	3.04E-07

[a] Standard error

[b] Total number of samples

[c] Number of samples with positive counts for the species

[d] p value

[e] adjusted p value

Fig. S2

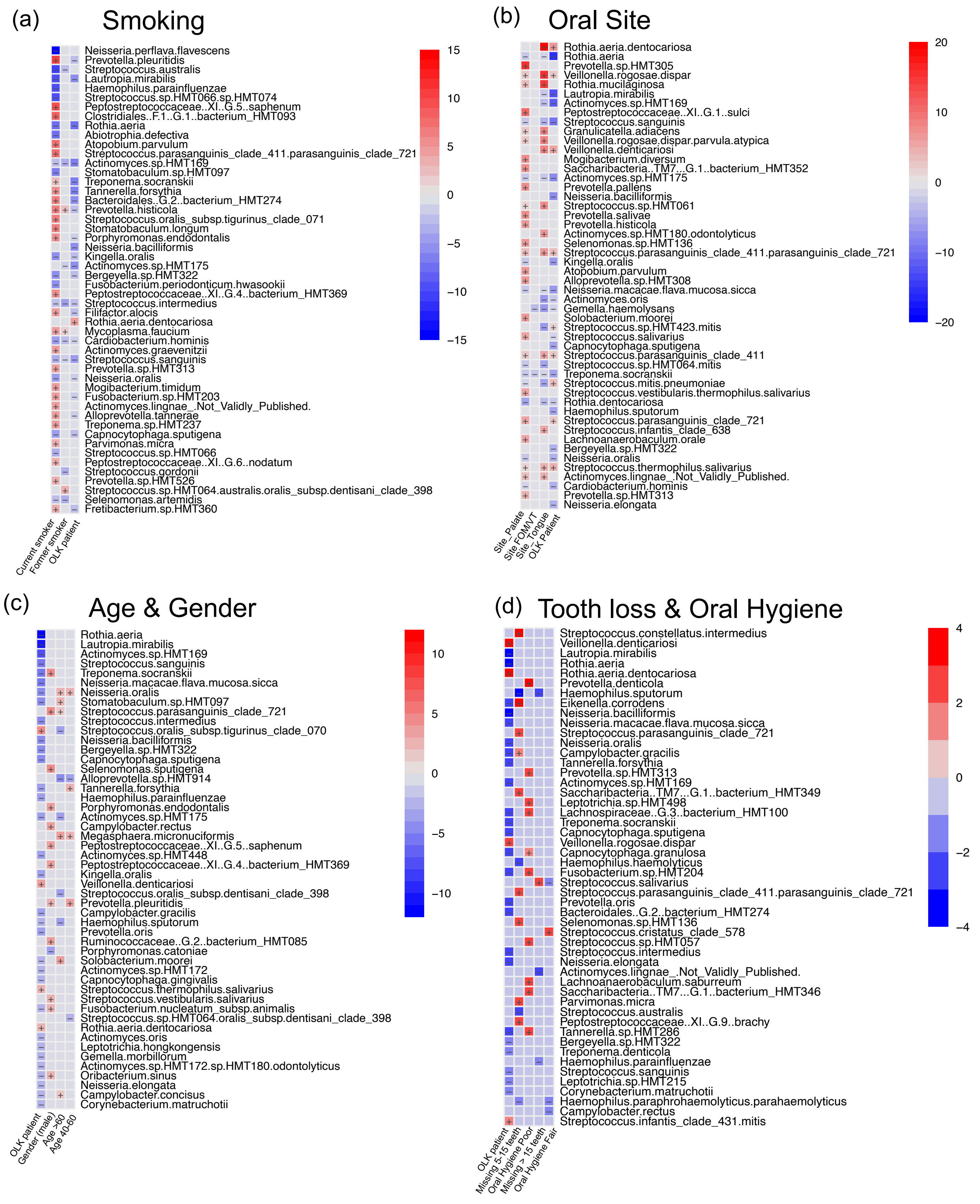

Fig. S2. Results of multivariate analysis of species abundance in healthy controls and OLK patients. Significant changes in species abundance in OLK patients (reference=healthy) are shown adjusted for (a) smoking (reference = non-smokers), (b) mucosal site (reference = buccal mucosa), (c) age (reference = <40) and gender (reference = female) and (d) tooth loss (reference missing <5 teeth) and oral hygiene (reference = good). Colour bar indicates significance calculated as -log (q-value) * (effect size). Red=increased, blue=decreased

Fig. S3

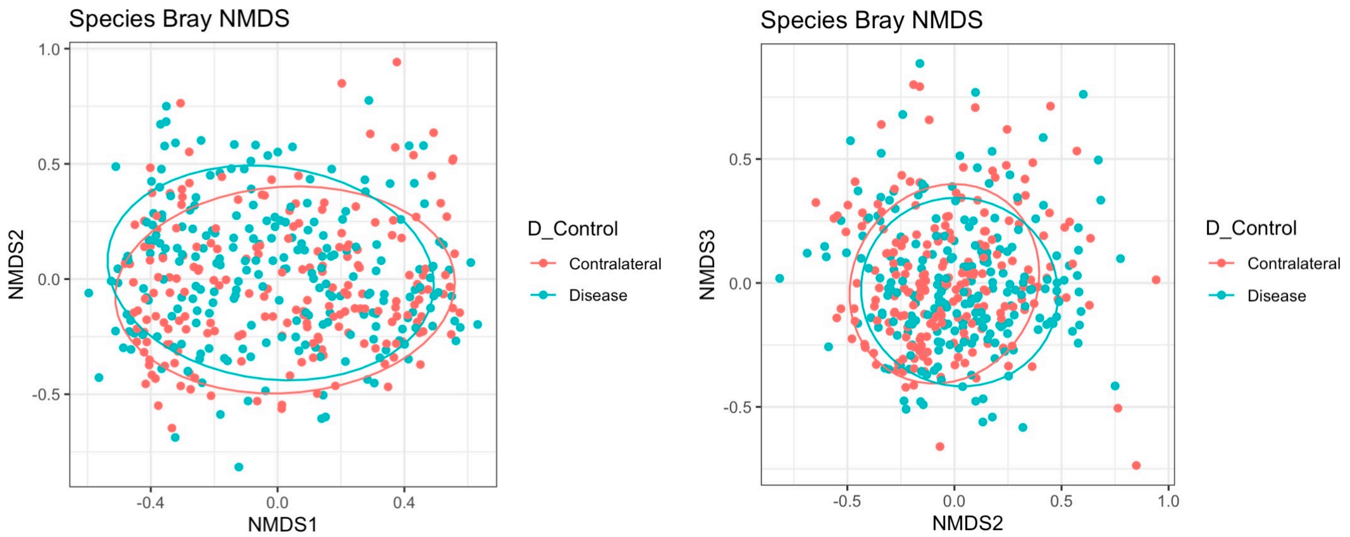

PERMANOVA p = 0.034

Fig. S3. Comparison of microbiome community structure in OLK and CLN sites from patients using non-metric multi-dimensional scaling (NMDS) analysis of Bray-Curtis dissimilarity values.

Fig. S4

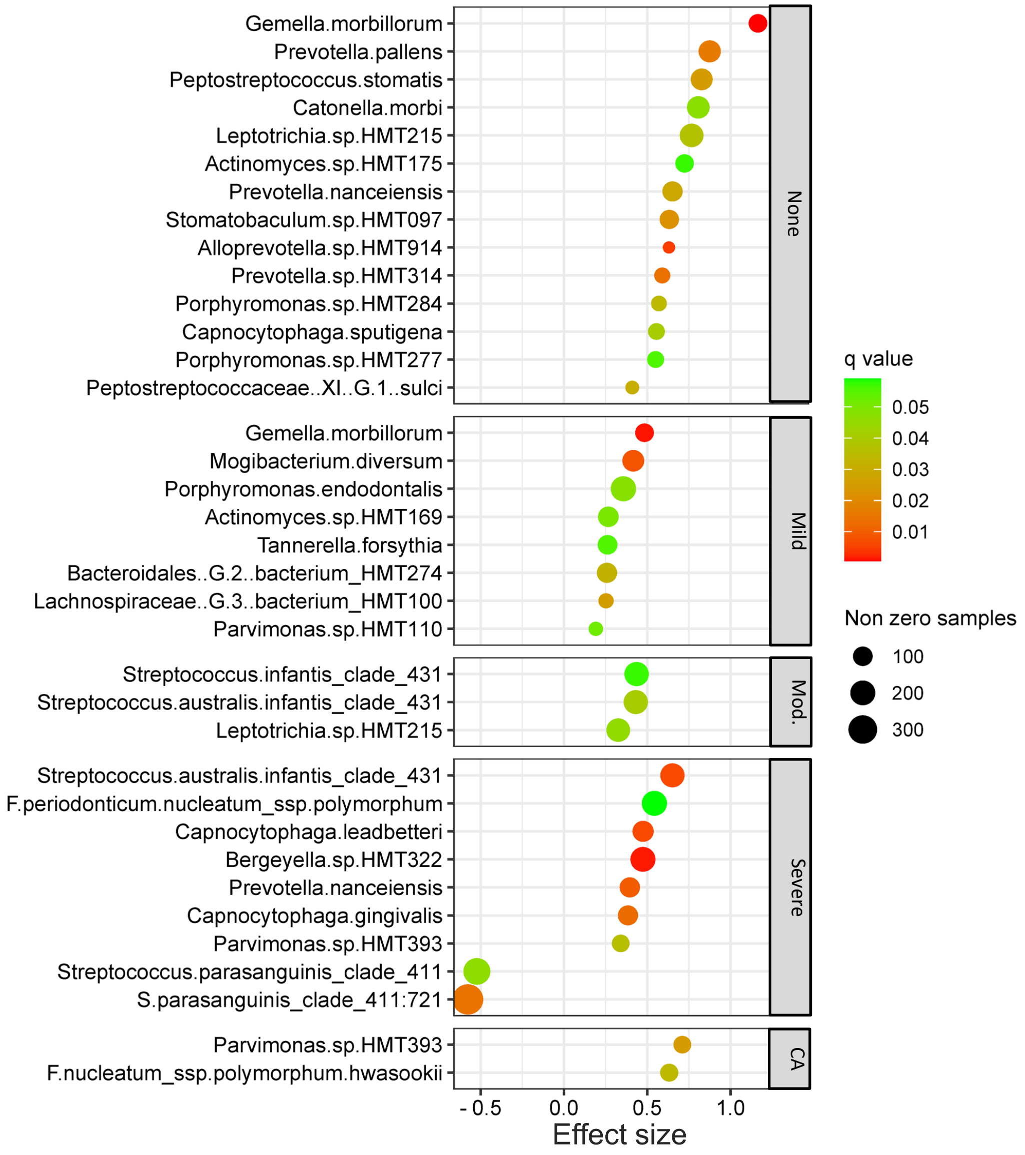

Fig. S4. Results of MaAsLin2 analysis to identify species significantly associated with none, mild. moderate or severe dysplasia. Symbols indicate level of significance (q values) and number of values (shape size).

Fig. S5

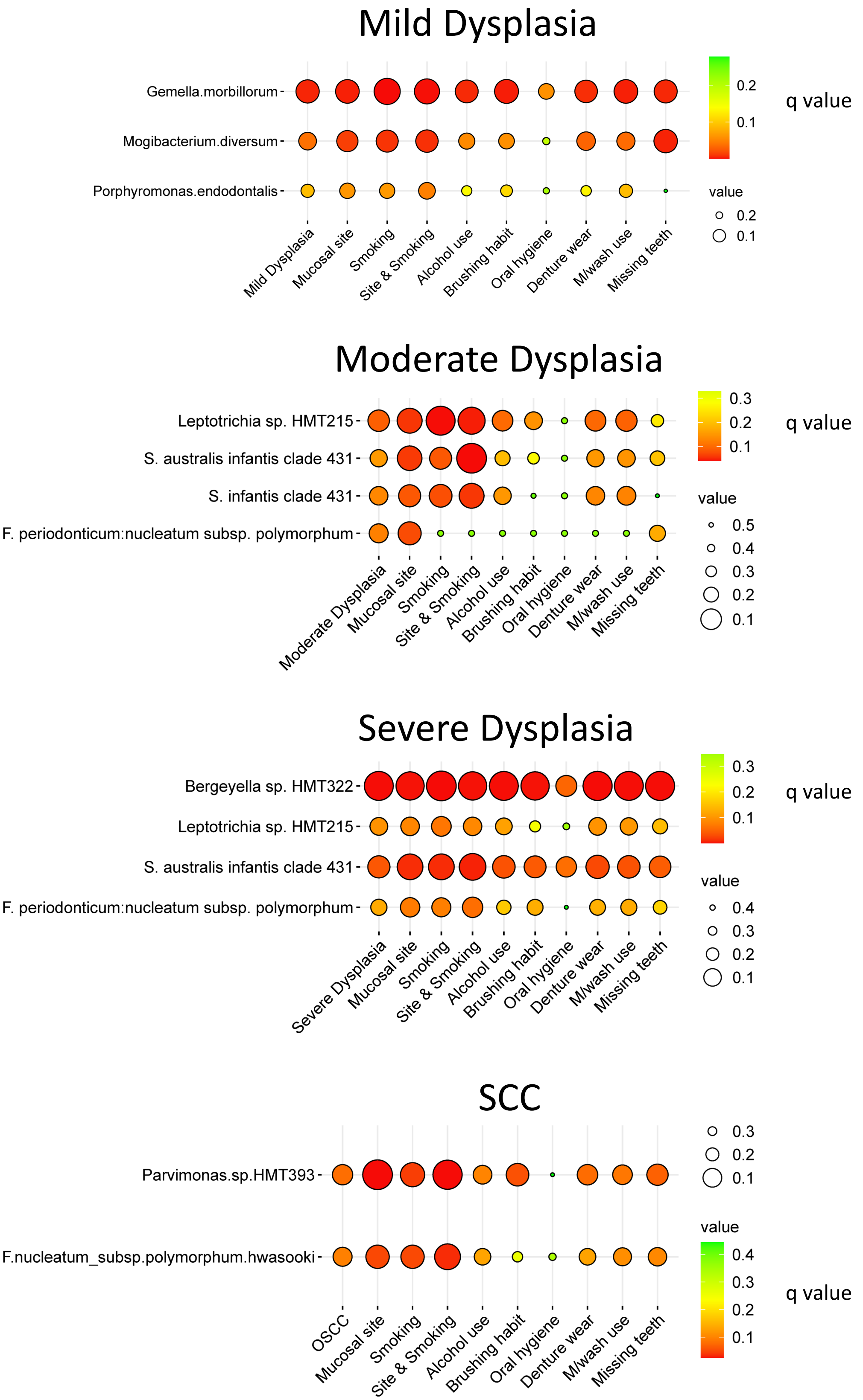

Fig. S5. Multivariate analysis of the microbiome of OLK exhibiting mild, moderate and severe dysplasia. Results of MaAsLin2 analysis are displayed showing the adjusted p value (q value) associated with each taxon tested for association with the degree of dysplasia and adjusted for the indicated patient variables.

Fig. S6

(a)

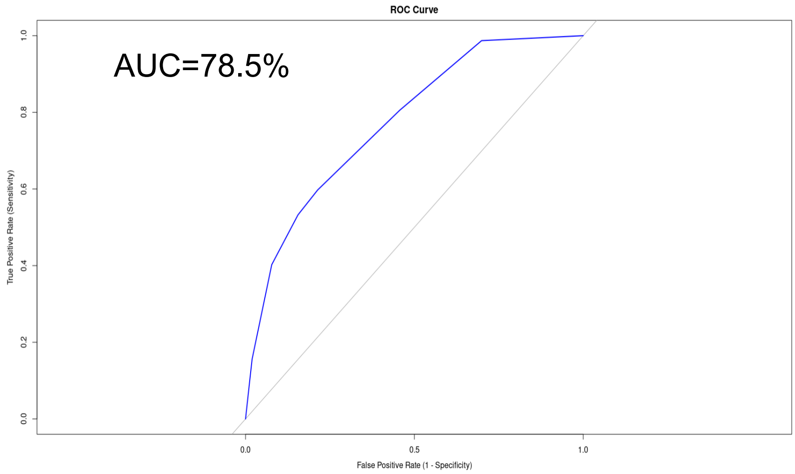

(b)

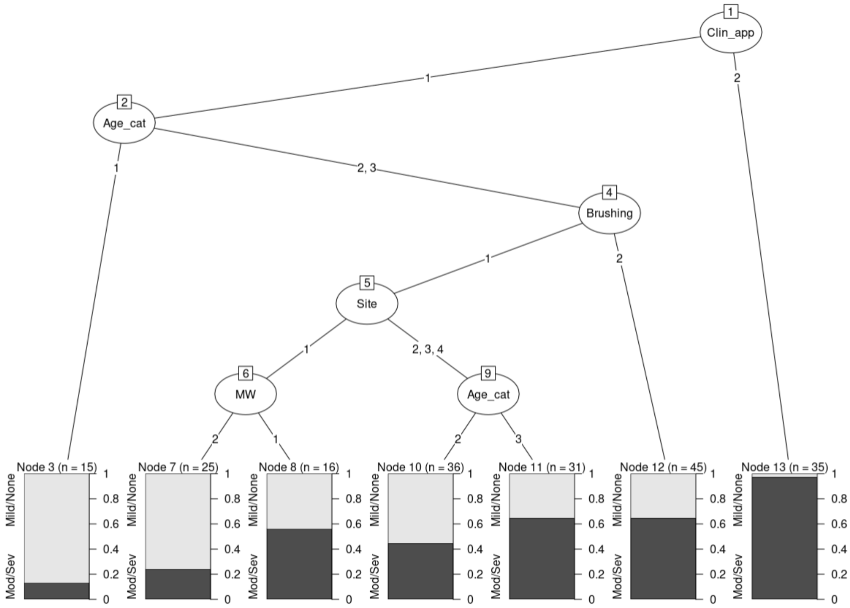

Fig. S6. Results of predictive modelling with randomForest using only clinical and demographic information to discriminate HROLK from LROLK. (a) Receiver operating characteristic curve for RF modelling using clinical and patient factors (AUC=78.5%). (b) Graphic of a Classification tree from RF modelling of clinical and patient factors. (Clin_app = clinical appearance, 1 = homogenous, 2 = non-homogenous; Age_cat = age category, 1 = <40 years, 2 = 40-60 years, 3 = >60 years, Brushing: 1 = More than once/day, 2 = once or less/day, Site 1 = Buc/Alv/Gin, 2 = FOM/VT, 3 = DT/LBT, 4 = PAL, MW = mouthwash use, 1 = Yes, 2 = No).

Fig. S7

(a)

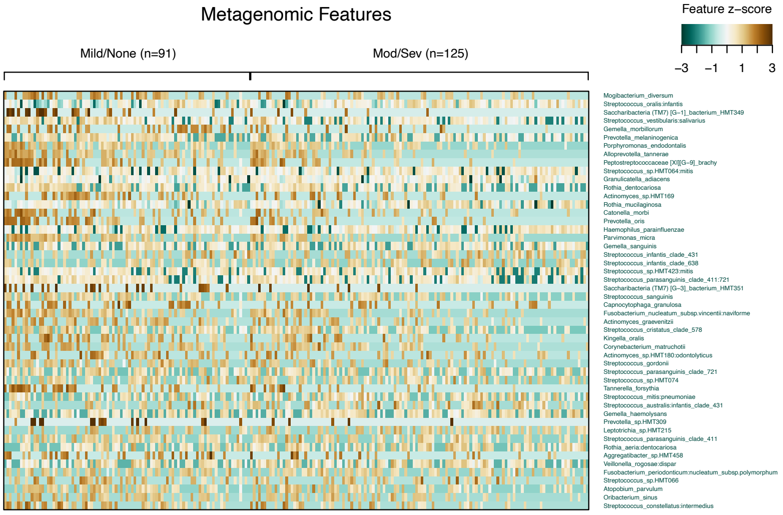

(b)

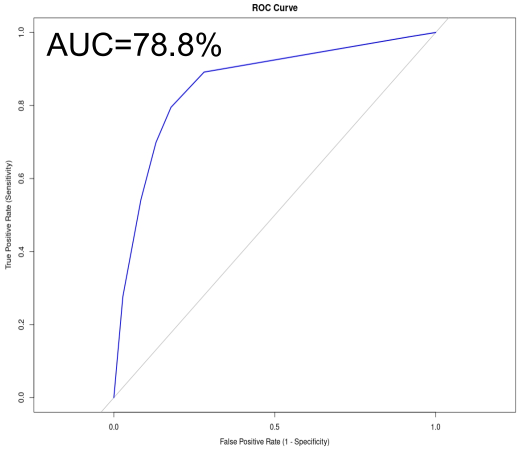

(c)

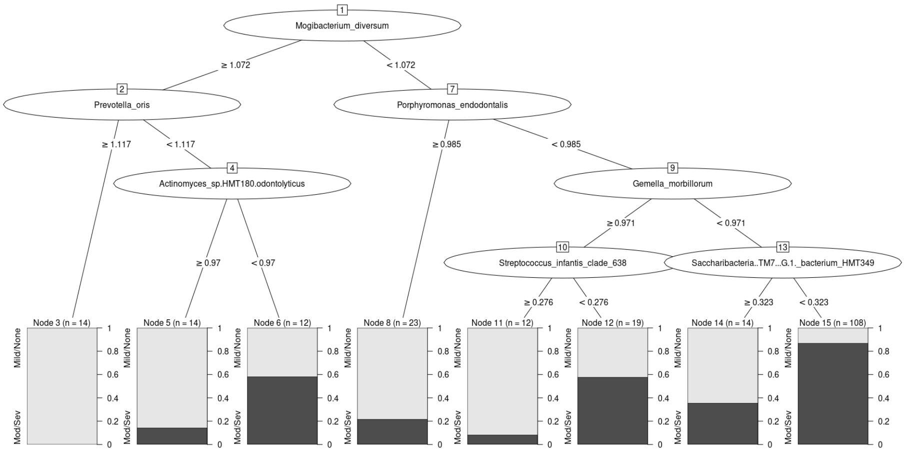

Fig. S7. (a) Heat map showing the z-scores of the 50 most discriminatory bacterial species identified by SIAMCAT. (b) Receiver operating characteristic curve for RF modelling using the z-scores of the top 50 microbial features in randomForest (AUC=78.8%). (c) Graphic of a Classification tree from RF modelling using the z-scores of the top 50 microbial features from initial SIAMCAT analysis.

Fig. S8

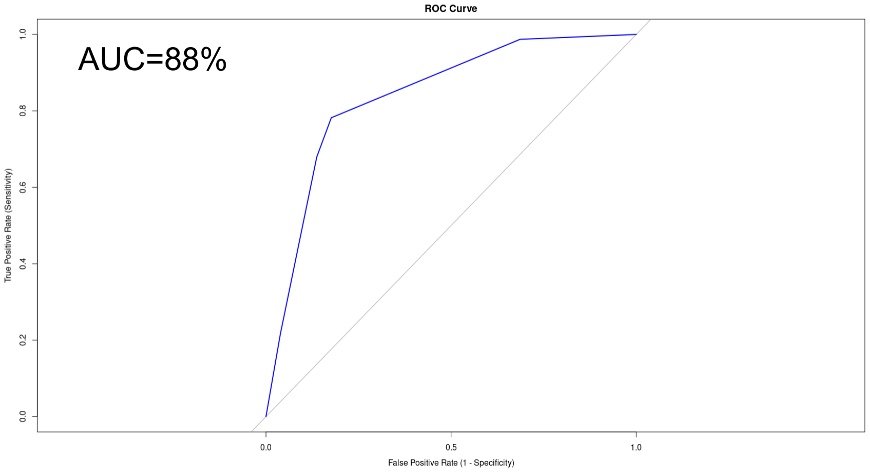

Fig. S8. Receiver operating characteristic curve for RF modelling using microbial features and patient data in randomForest (AUC=88%).

Table S3. Summary of predictive modelling results with randomForest to distinguish LROLK and HROLK using clinical and microbiome data alone and together .

	**Clinical[a]**	**Microbiome**	**Microbiome + Clinical[b]**
Sensitivity	76.3% (95%CI: 68.2%-84.3%)	85.5% (95%CI: 79.0%-92.0%)	87.4% (95%CI: 80.0%-93.6%)
Specificity	66.1% (95%CI: 55.8%-76.4%)	76.5% (95%CI: 67.3%-85.7%)	76.5% (95%CI: 67.2%-85.8%).
PPV[c]	75.5% (95%CI: 67.3%-83.8%)	83.5% (95%CI: 76.7%-90.3%)	83.4% (95%CI: 76.5%-90.4%),
NPV[d]	68.3% (95%CI: 57.6%-78.9%)	79.7% (95%CI: 70.8%-88.7%)	82.2% (95%CI: 73.5%-91.0%)
Accuracy	71.2% (95%CI: 64.6%-77.8%)	81.0% (95%CI: 75.4%-86.6)	81.9% (95%CI: 76.3-87.5%).

[a] Including all clinical parameters listed in Table S1 and data on oral site (Table 1).
[b] Including the most discriminatory clinical parameters shown in Fig. S7, namely clinical appearance, age category, mouthwash use, brushing frequency (Table S1) and oral site (Table 1).
[c] Positive predictive value
[d] Negative predictive value

Table S4. Levels of dysplasia in the main cohort and the repeat cohort of patients.

	**Main Cohort (n=226)**	**Repeat Cohort (n=107)**
**White Patches:**	216	103
• No Dysplasia	11 (5%)	3 (2.9%)
• Mild Dysplasia	80 (37%)	20 (19.4%)
• Moderate Dysplasia	76 (35%)	61 (59.2%)
• Severe Dysplasia	49 (23%)	19 (18.5%)
**OSCC**	10	4

Fig. S9

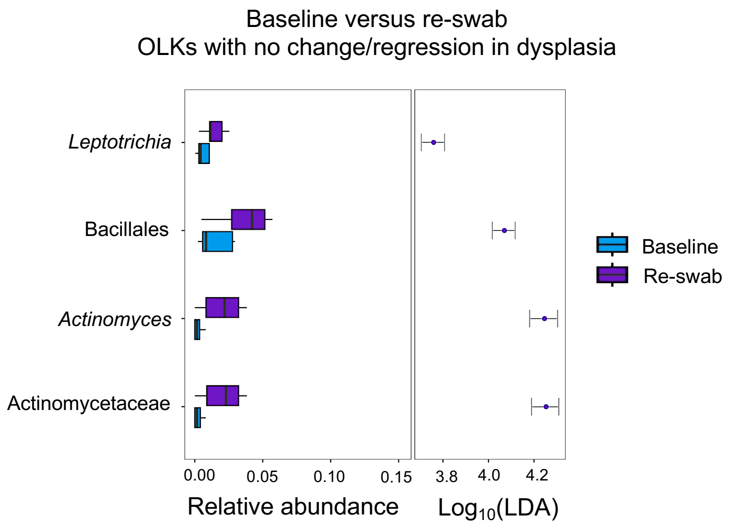

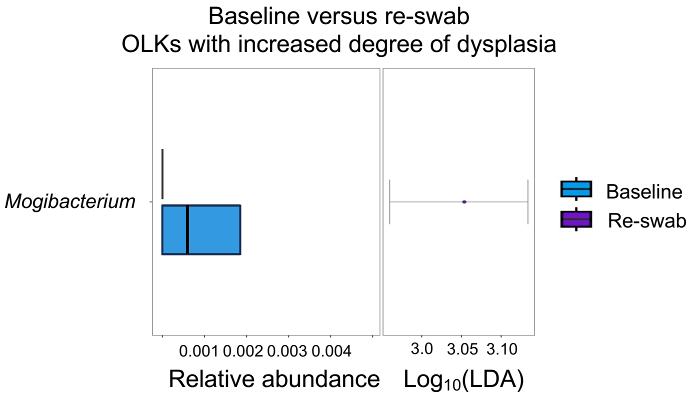

Fig. S9. Differences in species abundance identified between baseline and repeat swab samples in patients with no change or reduced dysplasia (No change/regression, top panel) and in those who exhibited an increased level of dysplasia (progression, bottom panel).
